# The COVID-19 vaccination acceptance/hesitancy rate and its determinants among healthcare workers of 91 Countries: A multicenter cross-sectional study

**DOI:** 10.17179/excli2021-4439

**Published:** 2022-01-06

**Authors:** Mehrdad Askarian, Aleksandr Semenov, Ferran Llopis, Francesca Rubulotta, Gorana Dragovac, Natalia Pshenichnaya, Ojan Assadian, Yvon Ruch, Zahra Shayan, Cristobal Padilla Fortunatti, Daniel Lucey, Abdullah Almohaizeie, Abu Hena Mostafa Kamal, Adenike Ogunshe, Aidos Konkayev, Asim Beg, Enzo Primerano, Fatma Amer, Hema Prakash Kumari Pilli, Ivan Hung, Folusakin Ayoade, Jean Yves Lefrant, Joanna Zajkowska, Jordi Rello, Momin Kazi, Mohammad Hossein Taghrir, Stijn Blot, Stephen Leib, Parisa Hosseinpour, Hamidreza Hosseinpour, Amirhossein Erfani, Roham Borazjani, Hossein Akbarialiabad, Masoud Najafi, Ardalan Askarian, Hakan Erdem

**Affiliations:** 1 Department of Community Medicine, School of Medicine, Shiraz University of Medical Sciences, Shiraz, Iran; Health Behavior Science Research Center, Shiraz University of Medical Sciences, Shiraz; 2 Ekaterinburg Research Institute of Viral Infections SRC VB Vector, Ekaterinburg, Russia; 3 Emergency Department. Bellvitge University Hospital, l'Hospitalet de Llobregat, Barcelona, Spain; 4 Department of Intensive Care Medicine, Charing Cross Hospital, Imperial College, NHS Trust London, UK; 5 Department of Epidemiology, Faculty of Medicine, University of Novi Sad, Serbia; Center of Disease Prevention and Control, Institute of Public Health of Vojvodina, Novi Sad, Serbia; 6 Clinical Department of Infectious Pathology, Central Research Institute of Epidemiology, Moscow, Russia; 7 Regional Hospital Wiener Neustadt, Austria; Institute of Skin Integrity and Infection Prevention, University of Huddersfield, UK; 8 Department of Infectious Diseases, Strasbourg University Hospital, Strasbourg, France; 9 Trauma Research Center, Department of Biostatistics, School of Medicine, Shiraz University of Medical Sciences, Shiraz, Iran; 10 School of Nursing, Pontificia Universidad Catolica de Chile, Santiago, Chile; 11 Department of Infectious Diseases, Georgetown University Medical Center, Washington, DC 20057, USA; 12 King Faisal Specialist Hospital and Research Center, Riyadh, Alfaisal University Riyadh, Saudi Arabia; 13 Department of Anaesthesiology & ICU, Rajshahi Medical College Hospital, Rajshahi, Bangladesh; 14 Applied Microbiology and Infectious Diseases, Department of Microbiology, Faculty of Science, University of Ibadan, Oyo State, Nigeria; 15 Department of Anesthesiology and Intensive Care, Astana Medical University, Nur-Sultan, Kazakhstan; National Centre of Traumatology and Orthopedia named by Batpenov, Nur-Sultan, Kazakhstan; 16 Department of Pathology and Laboratory Medicine, Aga Khan University, Karachi, Pakistan; 17 Department of Anesthesia and Intensive Care, Polyclinic of Monza, Monza, Italy; 18 Department of Medical Microbiology and Immunology, School of Medicine, Zagazig University, Zagazig, Egypt; 19 Department of Microbiology, GITAM Institute of Medical Sciences and Research, GITAM , Deemed to be University, Visakhapatnam, India; 20 Department of Infectious Diseases; Gastroenterology & Hepatology, The University of Hong Kong; 21 Division of Infectious Diseases, Department of Medicine, University of Miami Miller School of Medicine, Miami, FL 33136, USA; 22 Department of Anaesthesia, Critical Care Emergency and Pain Medicine, University Hospital of Nimes, Montpellier University, Nimes, France; 23 Department of Infectious Diseases and Neuroinfections, Medical University in Biaøystok, Poland; 24 CRIPS, Vall Hebron Institute of Research (VHIR) & CHRU Nimes, Nimes, France; 25 Research Department of Paediatrics and Child Health, Aga Khan University, Karachi, Pakistan; 26 Trauma Research Center, Shahid Rajaee (Emtiaz) Trauma Hospital, Shiraz University of Medical Sciences, Shiraz, Iran; 27 Department of Internal Medicine and Pediatrics, Ghent University, Ghent, Belgium; 28 Institute for Infectious Diseases, University of Bern, Bern, Switzerland; 29 School of Medicine, Islamic Azad University, Kazeroun branch, Kazeroun, Iran; 30 Department of Surgery, Shiraz Laparoscopic Research Center, Shiraz University of Medical Sciences, Shiraz, Iran; 31 Thoracic and Vascular Surgery Research Center, Shiraz University of Medical Sciences, Shiraz, Iran; 32 Student Research Committee, Shiraz University of Medical Sciences, Shiraz, Iran; 33 Student, College of Arts & Science, University of Saskatchewan, Saskatoon, Canada; 34 ID-IRI Lead Coordinator, Ankara, Turkey

**Keywords:** COVID-19, COVID-19 vaccines, Human Development Index, HDI, health personnel, vaccination coverage

## Abstract

The aim of this study was to investigate the COVID-19 vaccination acceptance rate and its determinants among healthcare workers in a multicenter study. This was a cross-sectional multi-center survey conducted from February 5 to April 29, 2021. The questionnaire consisted of 26 items in 6 subscales. The English version of the questionnaire was translated into seven languages and distributed through Google Forms using snowball sampling; a colleague in each country was responsible for the forward and backward translation, and also the distribution of the questionnaire. A forward stepwise logistic regression was utilized to explore the variables and questionnaire factors tied to the intention to COVID-19 vaccination. 4630 participants from 91 countries completed the questionnaire. According to the United Nations Development Program 2020, 43.6 % of participants were from low Human Development Index (HDI) regions, 48.3 % high and very high, and 8.1 % from medium. The overall vaccination hesitancy rate was 37 %. Three out of six factors of the questionnaire were significantly related to intention to the vaccination. While 'Perceived benefits of the COVID-19 vaccination' (OR: 3.82, p-value<0.001) and 'Prosocial norms' (OR: 5.18, p-value<0.001) were associated with vaccination acceptance, 'The vaccine safety/cost concerns' with OR: 3.52, p-value<0.001 was tied to vaccination hesitancy. Medical doctors and pharmacists were more willing to take the vaccine in comparison to others. Importantly, HDI with OR: 12.28, 95 % CI: 6.10-24.72 was a strong positive determinant of COVID-19 vaccination acceptance. This study highlighted the vaccination hesitancy rate of 37 % in our sample among HCWs. Increasing awareness regarding vaccination benefits, confronting the misinformation, and strengthening the prosocial norms would be the primary domains for maximizing the vaccination coverage. The study also showed that the HDI is strongly associated with the vaccination acceptance/hesitancy, in a way that those living in low HDI contexts are more hesitant to receive the vaccine.

## Introduction

The severe acute respiratory syndrome Coronavirus 2 (SARS-CoV-2), widely known as COVID-19, was first reported in December 2019 in Wuhan, China. The disease spread rapidly, infected the entire world and created a pandemic. As of the September 23, 2021 more than 230 million people in 223 countries/areas were affected by COVID-19 and caused more than 4.7 million deaths (Worldometer, 2021[24]). In addition to the effects on public health through morbidity and mortality, its detrimental impact on the economy has also been severe in many countries (Al-Mohaithef and Padhi, 2020[1]; McKibbin and Fernando, 2020[14]). Thus, taking necessary action to reduce the pandemic's consequences is a priority of health authorities.

Since the commencement of the pandemic, several approaches for preventing the spread of the novel coronavirus have been practiced, including mass quarantine, social distancing, wearing face masks, travel bans, and closure of schools and businesses. These mitigation strategies reduced the spread of the virus, however, were not always successful in flattening the epidemic curve and were not always a definitive solution (World Health Organization, 2020[21]). Moreover, despite all these efforts, the morbidity and mortality caused by COVID-19 often persist, and a heavy burden has been imposed on governments societies, businesses, and healthcare workers (Worldometer, 2021[24]). Thus, effective vaccination to provide active immunity is needed to help put an end to the pandemic (Lurie et al., 2020[13]).

Developing a safe and effective COVID-19 vaccine is a crucial challenge for scientists. Numerous researchers from both private and governmental sectors are working intensely to develop multiple reliable and effective vaccines (Callaway, 2020[3]). This process of testing and marketing new vaccines can take up to 15 years, however with the current critical pandemic situation, it has been reduced to around 12-18 months or even less (Krammer, 2020[11], Lurie et al., 2020[13]). Currently, around 200 types of vaccines are in different pre-clinical and clinical phases of development, which over 30 of them in clinical trials. For instance, Pfizer-BioNTech, Moderna's mRNA-1273 AstraZeneca/Oxford's AZD1222, China's Sinopharm and Sinovac, and Johnson and Johnson's one-dose adenovirus-vectored vaccine have received Emergency Use Listing (EUL) from the World Health Organization and are being administered throughout the world (Callaway, 2020[4]; Knoll and Wonodi, 2020[10]). Clearly, a successful vaccine's critical parameter is its efficacy, although the question is how much efficacy is essential to slow the pandemic. A minimum vaccine efficacy was set by the World Health Organization (WHO) as 50 %. When a suitable vaccine is available, it is essential to prioritize high-risk groups, including health care providers, those with comorbidities, immunocompromised, and the elderly (Askarian et al., 2021[2]; Sharma et al., 2020[17]).

Although vaccination can control the COVID-19 pandemic, low levels of trust with regard to COVID-19 vaccines can lead to vaccine hesitancy. The definition of vaccine hesitancy is "delay in acceptance or refusal of vaccination despite availability of vaccination services." The World Health Organization mentioned reluctance to receive vaccine despite of vaccine availability as one of the top ten health threats of 2019 (Dubé and MacDonald, 2020[7]; Pekmezci, 2019[16]). Many countries are dealing with anti-vaccine movements, lack of access to vaccines, as well as fighting the COVID-19 infodemic and the disease itself (Wilson and Wiysonge, 2020[22]).

Health care workers (HCWs) play an important role both on the frontlines of COVID-19 and also providing routine non-COVID-19 health services. Clearly, HCWs who have received vaccine, are better able to influence other person's willingness to take the COVID-19 vaccine (Nzaji et al., 2020[15]). A survey in China reported higher acceptance of COVID-19 vaccination among medical staff in comparison to general population. While another study in the United States showed only 20 % vaccine hesitancy among HCWs (Al-Mohaithef and Padhi, 2020[1]). Moreover, a survey in the Democratic Republic of the Congo (DRC) reported that nurses are more vaccine-hesitant than physicians and this hesitancy can negatively impact the rolling out of the COVID-19 vaccination (Nzaji et al., 2020[15]). Another study demonstrated several negative and positive predictors for vaccine inoculation, positive predictors include occupation as a physician, unemployment during quarantine period, working in health care service giving care to SARS-CoV2 positive patients, and negative predictors were working as a nurse and parenthood (Dror et al., 2020[6]).

Since the HCWs have a crucial role in this pandemic and have direct exposure to COVID-19, this multi-center survey was conducted to evaluate the acceptance of COVID-19 vaccine and its refusal rate among HCWs. The findings will help local and global health authorities in designing programs to maximize the vaccination coverage.

## Materials and Methods

This cross-sectional multi-center survey was conducted on an international scale between February 5 to April 29, 2021, to evaluate the COVID-19 vaccination acceptance/ hesitancy among HCWs and the factors that may contribute to it. The English version of the questionnaire was translated into seven languages including Persian, Russian, Italian, Spanish, German, French, and Serbian. The questionnaire was distributed through Google Forms using snowball sampling; a colleague in each country or context was responsible for the forward and backward translation, and also the distribution of the questionnaire. 

After participants entered the survey homepage, the aim of the survey and an online consent form were displayed. If the participants had an agreement to the survey objectives, they could officially start the survey by clicking the “Next” button below the form, or they could choose to cease the survey. Participation was entirely voluntary, non-commercial, and anonymous. Moreover, all investigators had signed confidentiality agreements before starting the study. The study was approved by the institutional board review of Shiraz University of Medical Sciences.

### Assessment and measures

The questionnaire consisted of three main sections. In the first section, participants were asked for some demographic information. This information included: age, gender, pregnancy status, current location, marital status, the highest level of education, history of chronic disease related to the severity of the COVID-19 (including diabetes mellitus, hypertension, lung diseases, renal diseases, cardiovascular diseases, and corticosteroids consumption), history of COVID-19 infection family history of COVID-19 infection, history of influenza vaccination, being a frontline HCW, current occupation, working in a public or private facility, and whether their health care facility admits COVID-19 patients or not.

As for the participants' location, we categorized them according to the World Bank classification of countries in the 2021 fiscal year for 'area' and 'level of income' (World Bank, 2020[23]), and 'Human Development Index (HDI)' based on the United Nations Development Program (UNDP) 2020 classification (United Nations Development Programme, 2020[20]). The human development index is a composite measure developed by the UNDP that aims at assessing the level of development of countries. The cutoff points were set as follow: HDI <0.550 for low human development, 0.550-0.699 for medium human development, 0.700-0.799 for high human development, and 0.800 or greater for very high human development.

The second section consisted of 26 items which were validated previously through factor analysis (questionnaire soon to be published in Int J Prev Med. 2022). These 26 Items were categorized into six factors encompassing: perceptions of the COVID-19 pandemic (three items), perceived benefits of the COVID-19 vaccination (four items), the vaccine safety/cost concerns (four items), preferences for alternatives (four items), prosocial norms (eight items), and risk reduction habits (three items). All items were answered in a 5-point Likert scale from 'Strongly agree' to 'Strongly disagree'. Items were scored from one for 'Strongly disagree' to five for 'Strongly agree' for all factors. A higher score indicates the higher willingness to take the vaccination except the items of vaccine safety/cost concerns and preferences for alternatives, where the higher score indicates higher hesitancy toward vaccination.

The third section was the primary outcome measure, willingness to receive COVID-19 vaccination. This section had one major item, "I will get the COVID-19 vaccine as soon as it is accessible". The answer was a 5-point Likert scale ranging from one ("strongly disagree") to five ("strongly agree"). For the analysis, we transform this 5-point scale to the binary view. 'Strongly agree' and 'Agree' were set as 'Yes' and the others as 'No/I don't know'. In addition, there was another item in this section that asked for the kind of vaccine they prefer (domestic, imported, or it makes no differences). 

Notably, the reliability of the whole questionnaire was calculated, and the Cronbach alpha was 0.89.

### Data analysis

Data were analyzed using SPSS version 16.0 (IBM, Armonk, NY, USA). Categorical variables were reported in number and percentage, and the Pearson Chi-square test was computed to detect the statistically significant differences between the demographics and the primary outcome; and the independent-sample t test was calculated for the continuous variables. A forward stepwise logistic regression was utilized to explore the variables and questionnaire factors that tied to the intention to COVID-19 vaccination. The two-tailed P-value<0.05 was set for the significance level.

### Ethical approval

Ethics approval of the study was obtained from the institutional review board of Shiraz University of Medical Sciences and Research Ethics Committee with the following numbers of 22654 and IR.sums.med.rec.1399.549, respectively. 

## Results

### Demographic information

Taken together, 4630 participants from 91 countries completed the questionnaire. The majority of participants were from Iran, followed by France, Kazakhstan, Italy, Spain, Saudi Arabia and another eighty-five countries. As far as the level of income is concerned, 2642 of participants (57.1 %) were from upper-middle-income countries, 39.6 % from high income, and only 3.3 % of the study population were from low and lower-middle countries. As for the region of participants, the main parts of participants were from the Middle East and North Africa (48.5 %), and Europe and Central Asia (41.8 %). Other regions are as follow: Latin America & the Caribbean (3 %), East Asia and Pacific (2.6 %), South Asia (1.5 %), Sub-Saharan Africa (1.2 %), and North America (1.4 %). According to UNDP, 43.6 % of participants were categorized into low HDI, 48.3 % high and very high, and 8.1 % medium.

In sum, the vaccination acceptance rate was 63 %, and 37 % of participants were hesitant to the COVID-19 vaccination. The mean age was 41.63 years; comparing the mean age between the outcome groups, it was significantly lower among those hesitant to receive the vaccine (P-value<0.001). Almost 66.8 % (3095) of participants were female; among them, 3 % were pregnant. Males were more willing to receive the vaccination.

Considering other demographics, roughly 70 % of participants were married, and 4 % considered their marital status as 'other' such as in-relationship (this concept might be different in different contexts). Those who were single were more hesitant to receive the vaccination. Doctorate or above and master's degree were the most self-reported highest level of education. Among the reported highest level of education, those with high school diploma the diploma and master's degree were less likely to accept the vaccination. Of all, 16.4 % mentioned that they have a chronic disease that would be related to the severity of COVID-19; and 28.3 % reported that they have had COVID-19 disease already. In addition, 81.9 % of participants had family history of the COVID-19 infection. Those who were not infected by SARS-CoV-2/COVID-19 had a higher willingness to receive the vaccination; parallel with those with negative family history of the COVID-19 infection. Almost two-thirds of participants had the influenza vaccine, which was strongly tied to their willingness to receive the COVID-19 vaccine.

As for occupation, medical doctors and nurses comprised the majority of the study population. Among them, medical doctors and pharmacists had significantly higher willingness to accept the vaccination. Notably, 85.6 % stated that their setting accepts and admits COVID-19 patients; and those caring for COVID-19 patients had a higher willingness to receive the vaccine. The participants also were asked their preference regarding the type of vaccine. Although 54.9 % stated that there is no difference between domestic or imported ones, 16.4 % responded that they only prefer domestic vaccines. Those who stated that there is no difference between the types of vaccine had a higher rate of vaccination acceptance. Other demographic characteristics are listed in Table 1(Tab. 1).

Since the vaccination acceptance was significantly different among male and female, and highest level of education, we did a subgroup analysis considering these two variables. Using Chi-square test, males with highest education level of Bachelor's, Master's, and Doctorate or above degree had higher vaccination acceptance rate compared to female. It might indicate that the significant difference among male and female in vaccination acceptance rate could be rooted in the highest level of education (Table 2(Tab. 2)).

Table 3(Tab. 3) outlined the mean scores of our six recruited factors of the questionnaire. Mean scores are shown separately between the outcome groups. All factors were significantly higher among those with more intention to receive the vaccine.

### Logistic regression

A forward stepwise logistic regression was performed to identify the significant factors related to the intention to receive the COVID-19 vaccine; the Nagelkerke R2 was 0.67. As shown in Table 4(Tab. 4), regarding the main questions, the following factors were significantly tied to higher intention to receive the vaccine: perceived benefits (Odds Ratio (OR): 3.82, 95 % Confidence Interval (CI): 3.23-4.51), safety/cost concerns, and prosocial norms (OR: 5.18, 95 % CI: 4.46-6.02). The factor of vaccine safety/cost concerns was also significantly related to our primary outcome (OR: 3.52, 95 % CI: 3.07-4.03), and because its items were scored inversely, the higher OR means higher hesitancy to the vaccination. As for demographic characteristics, the history of getting the COVID-19 was associated with vaccination hesitancy, and the history of receiving the influenza vaccine was also significantly related to the intention to receive the vaccine. Interestingly, HDI with OR: 12.28 (95 % CI: 6.10-24.72) was strongly associated with our participants' higher willingness to receive the COVID-19 vaccine. Other factors and demographics were not significantly related to vaccination intention.

## Discussion

Since the development of vaccines against SARS-CoV-2, several studies have been conducted concerning the vaccination acceptance among different populations. Here, we studied vaccination acceptance among healthcare providers in an international and multi-center survey. Our results demonstrated that doctors and pharmacist had higher rate of vaccination acceptance. Moreover, those who have never been infected with COVID-19 nor any of their family members, were more willing to receive the vaccine. Importantly, those who live in countries with higher HDI score had more willingness to be vaccinated than others.

HDI is defined as a summary measure of average achievement in key dimensions of human development: a long and healthy life, being knowledgeable and have a decent standard of living (United Nations Development Programme, 2020[20]). In a study by de Oliveira et al., HDI was shown to be a positive factor for vaccine dose per thousand persons, and countries with higher HDI received more doses of vaccine per thousand citizens (de Oliveira et al., 2021[5]). In our study, we sought to evaluate whether vaccine acceptance is also associated with higher HDI. The odds ratio was calculated to be 12.28, which means a high strong positive association of HDI with vaccine acceptance. This odds ratio was the highest rate we had in our study, showing its very important impact on vaccine acceptance. These findings were what we expected from countries with higher HDI. Since one of the primary aspects of calculating HDI is considering the knowledge domain, it is reasonable for countries with higher HDIs to have higher willingness to receive the vaccine. In a multinational study done in March 2020, it was found that those who live in countries with higher HDI and those who live in Europe were most aware of the importance of COVID-19 vaccination and its measure to implement receiving the vaccine (García-Toledano et al., 2021[9]). 

Among healthcare workers, medical doctors and pharmacists were more willing to receive the vaccine. However, working as a frontline healthcare worker was not statistically associated with vaccine acceptance. Since medical doctors and pharmacists usually work as the head of the teams in healthcare settings, they can increase awareness about beneficial effects of the vaccine administrations for their staff. In a study by Shekhar et al., vaccine acceptance was studied among healthcare workers in the United States (Shekhar et al., 2021[18]). They reported higher acceptance in healthcare workers involved in direct patient care and also workers with chronic medical conditions. In our study, those working as the frontline and those with history of chronic disease had higher vaccination acceptance rate, but the differences were not statistically significant. Furthermore, similar to our study, Dror and colleagues reported the highest level of vaccine hesitancy in nurses (Dror et al., 2020[6]).

Men were more likely to accept the COVID-19 vaccine. This was in line with previous studies (Solís Arce et al., 2021[19]; Wouters et al., 2021[25]). In addition, unmarried individuals were less willing to take the vaccine. This can be attributed to the fact that married people are more concerned about transmitting the disease to their family members, which is why they are more likely to receive the vaccine.

Regarding the main factors of our questionnaire, three of them have reached the level of statistical significance. In the factor of 'Perceived benefits of the COVID-19 vaccination' the participants were asked about the beneficial aspects of the COVID-19 vaccination. It contained four items including: 'If I know COVID-19 vaccination can protect me I'll do it', 'If I know COVID-19 vaccination can protect my family and my friends, I'll do it', 'If I know COVID-19 vaccination can protect other community members, I'll do it, and 'If I know COVID-19 vaccination can help return society to normal public to normalcy, I'll do it'. According to the logistic regression model, its odds ratio was 3.82, signifying a positive association with vaccine acceptance. 

The odds ratio of 'The vaccine safety/cost concerns' was 3.52. Since its items were scored inversely, this odds ratio means that this factor was positively associated with vaccine hesitancy. The items were as follow: 'I'm concerned about potential side effects of COVID-19 vaccine', 'I think COVID-19 vaccine may not be safe', 'I have concerns about getting COVID-19 from the vaccine', and 'Vaccination may have some cost for me'. Previous studies reported similar results; they reported that the fear of potential side effects was the most common reason for vaccine refusal (Elharake et al., 2021[8]). However, as more people get vaccinated, more accurate information becomes available about the effectiveness and possible side effects of vaccines which may lead to more vaccine acceptance over time.

In the factor of 'Prosocial norms', the HCWs were assessed through eight items. The domain of items consisted of the role of national TV programs, social media, trusted physician, parents, and friends in convincing them to receive the vaccine. The odds ratio was 5.18 which was strongly tied to vaccination acceptance.

The history of contracting COVID-19 disease, and also infection in family members, were positively associated with vaccine hesitancy. Also, the odds ratio for the history of COVID-19 disease was 0.76, which is tied to vaccine hesitancy. Maybe this was because these individuals thought they were immune against COVID-19 after contracting it or because they thought that they once defeated COVID-19, they could still do it. Besides, individuals with a positive history of flu vaccination were more willing to receive the COVID-19 vaccine, similar to previous studies (Shekhar et al., 2021[18]). Also, the odds ratio of the history of flu vaccination was 1.35. As expected, it shows that people who accept the concept of vaccination and also, people who care more about their health were willing to take COVID-19 vaccines.

In regard to educational level, bachelor's degree and associate degree were more eager to receive the vaccine, in contrast to individuals with diplomas and master's degrees who were vaccine-hesitant. So, we did not observe an increase in the desire to receive the vaccine from the lowest to the highest educations level in our study. A multinational survey study done by Lazarus et al. demonstrated various result based on countries and educational level in acceptance of COVID-19 vaccine. Higher educated people in the United States, Ecuador, France, Germany and India were more likely to be vaccinated while higher educated ones in Spain, Canada and United Kingdom were more vaccine hesitant (Lazarus et al., 2020[12]).

## Conclusion

This study highlighted the vaccination hesitancy rate of 37 % in our sample among HCWs. Increasing awareness regarding benefits of the vaccination, confronting the misinformation, and strengthening the prosocial norms would be the primary domains for maximizing the vaccination coverage. The study also showed that the HDI is strongly associated with the vaccination acceptance/hesitancy, in a way that those living in low HDI contexts are more hesitant to receive the vaccine.

## Declaration

### Acknowledgment

We would like to thank Prof. Daniel R. Lucey for his great contributions in reviewing and editing the paper.

### Conflict of interest

The authors declare that they have no known competing financial interests or personal relationships that could have appeared to influence the work reported in this paper.

## Figures and Tables

**Table 1:**
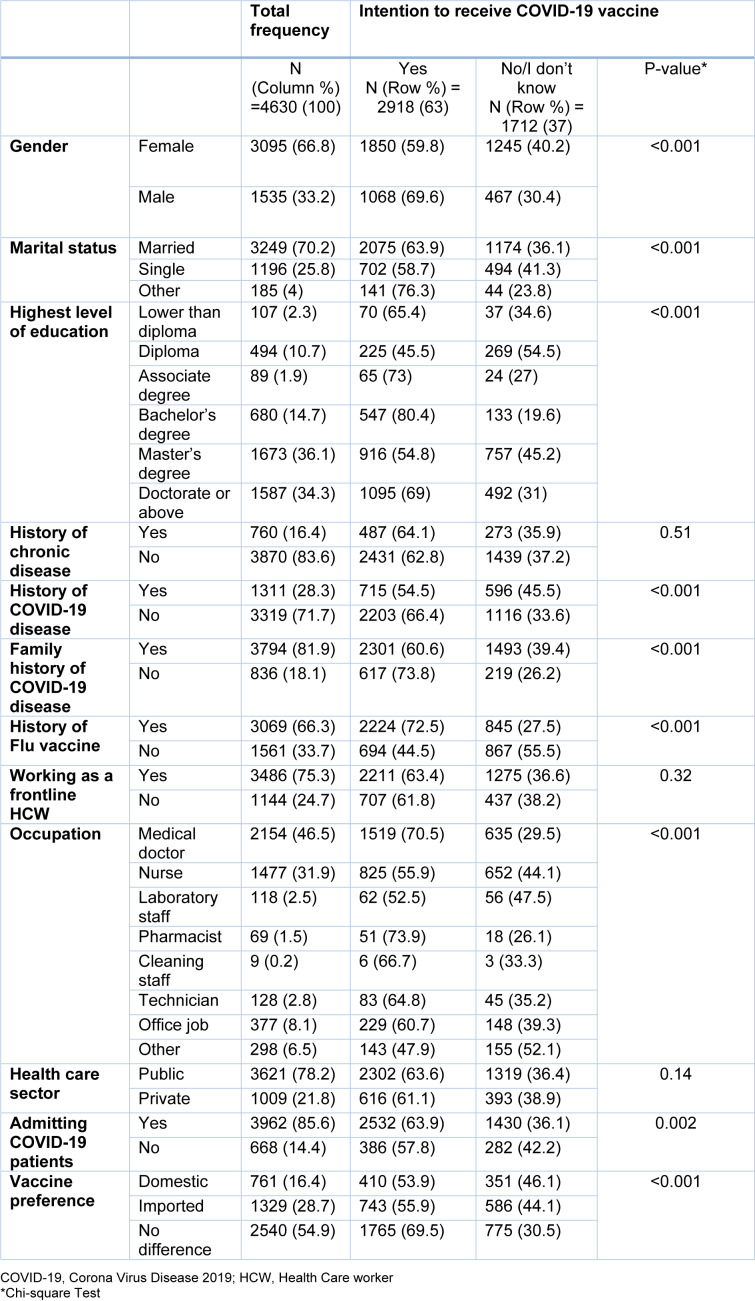
Demographic variables and their association with the primary outcome

**Table 2:**
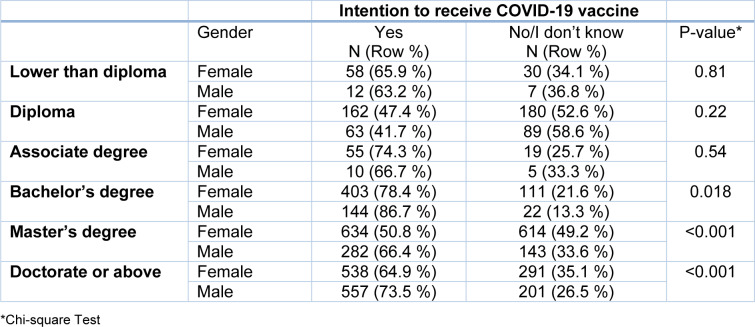
Sub-group analysis considering Gender and Highest level of education

**Table 3:**
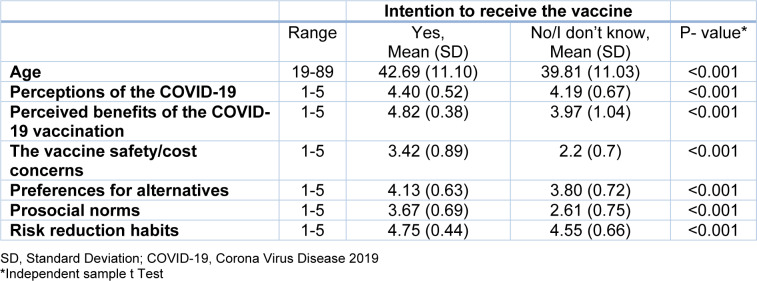
The details of continuous variables and their association with the primary outcome

**Table 4:**
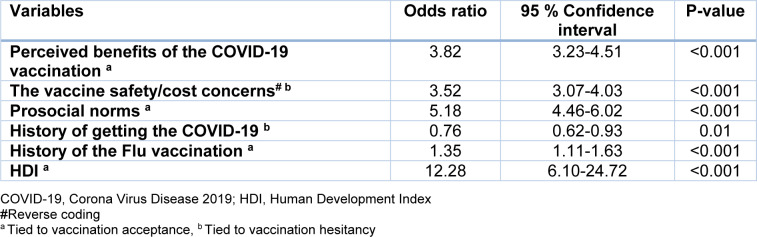
The results of the forward stepwise logistic regression
